# Long-Term Outcome after Resection Rectopexy for Internal Rectal Intussusception

**DOI:** 10.5402/2012/824671

**Published:** 2012-12-30

**Authors:** Egil Johnson, Kristin Kjellevold, Hans-Olaf Johannessen, Anders Drolsum

**Affiliations:** ^1^Department of Gastroenterological and Pediatric Surgery, Oslo University Hospital, Ulleval, Kirkeveien 166, 0407 Oslo, Norway; ^2^Faculty of Medicine, University of Oslo, P.O. Box 1072 Blindern, 0316 Oslo, Norway; ^3^Department of Radiology, Oslo University Hospital, Ulleval, Kirkeveien 166, 0407 Oslo, Norway

## Abstract

*Background and Aims*. The optimal treatment of patients with internal rectal intussusception (IRI) is unresolved. The aim was to study the short- and long-term outcome of resection rectopexy in these patients. *Methods*. An observational and mainly prospective study of 48 patients (44 women) with IRI who had ligament-preserving suture rectopexy by laparoscopic (*n* = 25) or open (*n* = 23) technique. Outcome measures were morbidity, scores for constipation and anal incontinence, patients' report, and health-related quality of life (HRQoL). *Results*. From preoperatively to a median of 6 months and 76 months postoperatively, constipation scores were reduced from a mean of (95% CI) 13.20 (11.41 to 15.00) to 6.91 (5.29 to 8.54) and 6.35 (4.94 to 7.76) (*P* < 0.0001). The number of constipated patients was reduced from 35 to eleven and eight, respectively, and none became constipated. Nine of ten symptoms of constipation improved. Corresponding scores for anal incontinence were 4.7 (2.4–7.0), 4.0 (2.4–5.7), and 4.1 (2.3–5.8), respectively. HRQoL at long-term followup compared to the general Norwegian population was reduced in four out of eight dimensions concerning physical factors. The patient-reported outcome at short- and long-term followup was improved by 85.4% and 75.0%, respectively. *Conclusions*. Resection rectopexy for IRI improved the outcome. HRQoL was reduced compared with the general population.

## 1. Introduction

Internal rectal intussusception (IRI), usually occurring in women, is a circumferential invagination of the entire rectal wall that may reach the anal canal. Both anoscopy and defecography are necessary for confirmation of the diagnosis in straining patients. Typical concomitant findings are rectocele and enterocele [1]. A minority of patients with demonstrable IRI and other findings at defecography may present without symptoms of defecatory disorder [2, 3]. Prevalent symptoms in afflicted patients are constipation including incomplete rectal evacuation of stool, pain, and fecal incontinence [1, 4–8]. 

An established indication for surgery has been fecal incontinence [4, 7, 9, 10] which usually improved after transabdominal rectopexy. On the other hand, incomplete rectal evacuation and constipation may be worsened [4, 7, 8, 10, 11] by this method. In patients with overt rectal prolapse, sigmoid resection in addition to rectopexy (resection rectopexy) may reduce constipation [12–14]. 

From 2000 to 2006, four studies [15–18], using open or laparoscopic resection rectopexy for IRI, reported improvement in both anal incontinence and constipation in these patients. A clue for the adequate evaluation of these patients is to identify constipation correctly [12, 13, 19–21]. Recently, constipation has been more clearly classified using linear discriminant analysis of 10 graded symptoms in the so-called KESS questionnaire (Knowles-Eccersley-Scott-Symptom questionnaire) [22].

Using this scoring system to define constipation, we examined whether the use of resection rectopexy could improve the symptomatic outcome in patients with IRI at short-term and long-term followup. Outcome also comprised complications, patient satisfaction, and health-related quality of life (HRQoL).

## 2. Materials and Methods

### 2.1. Patients

Fifty-three patients with IRI had resection rectopexy between February 1999 and June 2006. One patient died from malignancy and had sigmoidostomy for anal incontinence. Three patients were excluded from followup because of not responding (*n* = 3). Accordingly, 48 of the initial 53 patients (90.6%) were evaluated in this study (Table 1). A prerequisite for being considered for surgery was heavy clinical symptoms (constipation and/or anal incontinence), refractory to conservative treatment, and demonstration of IRI both by defecography and anoscopy. In 47 patients, there was a full wall circular intussusception more than 3 mm thick reported to be symptomatic [23]. In one difficult case the degree of intussusception was concluded most likely to be a complete full wall internal prolapse.

Twelve patients (25%) had undergone previous surgery: hysterectomy (*n* = 4), salpingo-oophorectomy (*n* = 1), colonic resection (*n* = 2), stapled transanal mucosal resection (*n* = 1) and operations for lumbar disc prolapse (*n* = 2), genital descent (*n* = 1), and rectocele (*n* = 1). Two patients developed constipation shortly after operation for lumbar disc prolapse [24, 25], two had persisting spastic anus, and one woman was anorectic. Thirty-four of the 44 (77.3%) women had had vaginal delivery.

### 2.2. Surgical Procedure

Rectopexy [26] with suture and sigmoid resection was performed [16]. Rectum was mobilized posteriorly in the mesorectal plane to the tip of coccyx. Anteriorly the dissection was kept close to the rectal wall to the junction of the upper and middle third of vagina or to the seminal vesicles, preserving most of the lateral ligaments. The mesorectum was fixed loosely in the midline posteriorly with usually two absorbable sutures to the presacral fascia 2–4 cm below the promontory. It aimed at avoiding tension on the rectum, which followed the sacral curve. Redundant sigmoid was resected, and an anastomosis was hand sewn with one continuous seromuscular suture in the open operations and with a stapled end-to-end anastomosis in the laparoscopically assisted operations. The operation was done laparoscopically in 25 patients and by open access in 23 patients, including conversions to open operation in three patients. Reasons for conversions were adhesions, rectal perforation by staple gun, adhesions, and rectal stricture necessitating a stapled end-to-side sigmoidorectostomy.

### 2.3. Followup

The patients were examined in the outpatient clinic preoperatively and at short-term followup (Table 1), mainly by two consultants involved in their treatment. At long-term followup the patients were evaluated by phone interview by an unfamiliar trainee. Symptoms and the patients' report on outcome of treatment were recorded. Fecal incontinence was assessed by St. Marks score [27]. Perfect continence to total incontinence is represented by a minimum score of 0 to a maximum score of 24 points, respectively. In 12 patients, assumed to be particularly constipated, colonic transit time was measured in median of 10.5 months (range 6–62 months) before and median of 7.5 months (range 2–17 moths) after operation, respectively. More than 4.2 days was diagnosed as slow transit [28]. From January 2001 the validated KESS score [22] was used, which contains 10 questions designed to define constipation. The total score was the sum of the scores for each question with a maximum possible score of 35 points. Using a cut-off criterion of ≥10 points, the KESS score discriminated between constipated patients and healthy controls with a sensitivity of 100% (95% confidence interval (CI) 95–100%) and a specificity of 100% (63–100%). The KESS questionnaire was answered prospectively, except from retrospectively by initial 14 patients concerning preoperative symptoms. At long-term followup patients were also asked about health-related quality of life (HRQoL). The patients had postoperative defecography at short-term followup, of whom 18 also volunteered at long-term followup. 

HRQoL was assessed with the short form 36 (SF-36) (version 2) generic questionnaire consisting of 36 items. Thirty-five of these are grouped into the following eight health domains: (1) physical functioning, (2) social functioning, (3) role limitations due to physical problems, (4) role limitation due to emotional problems, (5) mental health, (6) vitality (energy and fatigue), (7) bodily pain, and (8) general health perception. Each domain is graded on a scale of 0–100, and the higher the score the better the HRQoL. The validity and reliability of the SF-36 form have been demonstrated for a number of countries including Norway (version 1) [29]. The data were compared with published norms from 2323 individuals in the general population. Although there are differences in the grading of some questions in version 2 versus version 1 of the SF-36 questionnaire for the four health dimensions 3, 4, 5, and 6, the mean values on a group level are comparable.

### 2.4. Statistical Analysis

McNemar test was used to compare within-group changes in the proportion of symptoms or findings after operation. Differences in constipation score and incontinence score from pre- to postoperatively were assessed with parametric repeated measures analysis of variance (ANOVA) with Bonferroni multiple comparisons test using the Instat for Windows statistics software package (Graphpad Software, San Diego, CA, USA). Paired *t*-test was used to examine colonic transit time as a consequence of operation. Comparison of HRQoL between the patients and the background population was performed using unpaired *t*-test with Welch correction, based on the assumption that the populations have different standard deviations. Probabilities of less than 0.05 were considered significant.

### 2.5. Ethical Considerations

The short-term followup of the patients was considered as routine clinical practice. The regional ethical committee approved the long-term followup of these patients who consented.

## 3. Results

### 3.1. Morbidity

Eight patients (16.7%) had major early morbidity. In open surgery, four patients had partial transection of ureter, reoperations for bleeding, or percutaneously drained intra-abdominal abscess. In laparoscopic surgery, four patients had rectal perforation necessitating temporary ostomy, incarcerated hernia, hematoma from trocar-induced lesion of inferior epigastric artery, or lung embolus. Three patients (6.3%) had delayed morbidity with operations for incisional hernia, intestinal obstruction, and anastomotic stricture including a temporary ostomy.

Four patients (8.3%) had additional surgery in order to improve outcome, of whom three had operations for proctocele and transanal stapled resection of mucosal prolapse (*n* = 2). One patient with a persisting enterocele had at defecation pain and incomplete evacuation of stool, that was relieved by stretching close to supine position. She underwent a laparoscopic suture of anterior rectum to anterior pelvic peritoneum that removed the enterocele and relieved her symptoms. 

 A fifth patient improved from increased constipation after resection rectopexy, by sacral nerve stimulation.

### 3.2. Defecography

At preoperative defecography performed in all 48 patients included in this study, 34 (70.8%) and 14 patients (29.2%) had a moderate and deep intussusception reaching into the middle or deep part of rectum, respectively. In 46 of these patients defecography was also performed at short-term followup (Table 2). Intussusception was removed in 42 patients, unaltered in one, and reduced in size in three patients, respectively. There was a reduction in number of rectocele (*P* = 0.02), and their size diminished in 6 of the patients. Rectal motility, rather than evacuatory capacity, was also close to significantly decreased (*P* = 0.05).

 Eighteen of the 46 patients (39.1%) also had defecography at long-term followup after median 97 months (range 58–132 months), of whom recurrent IRI was detected in two patients (11.1%). Persisting but smaller intussusceptions in three patients were no longer demonstrated, of whom one had had a transanal mucosal resection. The corresponding number of patients with reduced rectal motility declined from three to one, respectively. 

### 3.3. Constipation

Compared with preoperatively constipation scores were similarly reduced at short- and long-term followup from (mean and 95% CI) 13.20 (11.41–15.00) to 6.91 (5.29–8.54) and 6.35 (4.94–7.76) (*P* < 0.0001). The corresponding number of constipated patients (score ≥ 10) was 35, 11, and 8, respectively. Thirteen patients without initial constipation (score < 10) remained devoid of this symptom. Two constipated patients had increased scores at short- (*n* = 1) and long-term followup (*n* = 2). Nine of 10 KESS score symptoms improved at short-term and long-term followup, respectively (Table 3). Stool consistency was not significantly improved, neither at short- nor at long-term followup. Minutes in lavatory per attempt, painful evacuation effort, and frequency of bowel movement improved, whilst laxative use deteriorated from short- to long-term assessment, respectively. Most frequent symptoms were feeling incomplete evacuation of stool (95.8%), bloating (81.3%), minutes in lavatory per attempt (75.0%), and abdominal pain (70.8%). Symptoms most strongly removed were use of enemas/digitation, painful evacuation effort, and unsuccessful evacuatory attempts, respectively. The KESS questionnaire answered retrospectively by 14 patients concerning preoperative data showed no significant differences (data not shown) in scores preoperatively or postoperatively compared with remaining 34 patients, exclusively prospectively evaluated.

### 3.4. Incontinence

Anal incontinence scores from preoperatively to short-term and long-term followup were mean (95% CI) 4.7 (2.4–7.0), 4.0 (2.4–5.7), and 4.1 (2.3–5.8), respectively. Corresponding number of patients with varying degree of anal incontinence (score ≥ 2) was 15 (31.3%), 21 (43.8%), and 22 (45.8%). At short-term and long-term followup incontinence was removed in four and nine, reduced in seven and four, unaltered in four and three, increased in zero and five, and occurred in ten and ten patients, respectively. Generally, major anal incontinence was reduced from preoperatively to both short-term and long-term followup, whilst a simultaneous slight increase of patients with minor leakage occurred (Table 4).

### 3.5. Transit Time

Mean (95% CI) colonic transit times before and after operation in 12 constipated patients were 4.8 (3.6–6.0) and 3.8 (2.6–4.9) days (*P* = 0.12). Seven and four patients had slow transit preoperatively (>4.2 days) and postoperatively, respectively, of whom one developed slow transit after operation. Colonic transit time was reduced in nine, unaltered in one, and increased in two patient(s), respectively. Initial constipation (score ≥ 10) was at long term removed in 10 of these patients, who also reported an improved treatment result. 

### 3.6. Patients' Report

Forty-one patients (85.4%) and 36 patients (75.0%) reported an improved treatment result at short- and long-term followup (Table 5), respectively. The six patients with unaltered results at long-term had a general reduction of constipation scores but relatively stable incontinence scores. Moreover, two of these patients had either anismus (*n* = 1) or mucosal prolapse (*n* = 1). Five of six patients with worse results at long-term followup had persistent constipation, of whom two had moderate and considerable incontinence, respectively. The sixth patient had a minimal reduction of constipation score (from 10 to 8). Interestingly, six of the 12 patients with unaltered or deteriorated long-term result had had complications and need for additional procedures.

### 3.7. Subgroups

We demonstrated no significant differences in constipation scores preoperatively and at short term and long term postoperatively between the following subgroups; laparoscopic (*n* = 25) and open technique (*n* = 23), moderate (*n* = 34) and deep rectal intussusception (*n* = 14), and without (*n* = 33) and with (*n* = 15) complications and additional surgery. Although not significant, there was at long-term followup a trend towards improved constipation score (8.1 (4.7–11.3) versus 5.6 (4.1–7.0), *P* = 0.15) in the group without complications. This potential difference was also supported by the patient-reported improved treatment in 60.0% versus 84.6%, respectively. 

### 3.8. Health-Related Quality of Life

Because of a limited number of patients (*n* = 48), of whom four were men, we chose to report long-term HRQoL not adjusted for gender and age (Table 6). The patients operated for IRI scored significantly lower than the general population for the four dimensions general health perception, bodily pain, physical functioning, and role limitation due to physical problems. On the other hand, health dimensions related more strongly to psychological factors like vitality, mental health, social functioning, and role limitation due to emotional problems were comparable with the general population.

## 4. Discussion

The main finding in this study of resection rectopexy for IRI was that the improved outcome mainly persisted from short-term to long-term followup. 

Contrary to other studies [15, 17, 18] using resection rectopexy for rectal intussusception, we have used [16] the graded score KESS score [22], for determination of constipation. Accordingly, both removal and alteration of constipation based on 10 different symptoms can be detected in these patients. The broad definition of constipation made possible by this score also seemed more adequate than more restricted interpretations based on disturbed colonic motor activity or outlet obstruction [12, 13, 19, 20]. Thus, both the symptoms most prevalent (feeling incomplete evacuation, bloating, minutes in lavatory per attempt, and abdominal pain) and the symptoms with the strongest improvement (enemas/digitation, unsuccessful evacuatory attempts, and painful evacuation effort) can be identified (Table 3). It was also comforting to observe that use of enemas and digitation for emptying of stool was considerably reduced. Because the KESS score was introduced in 2000, initial symptoms in the first 14 patients in this study were scored at short term postoperatively, which represented a source of bias. However, this problem was negligible since biased and unbiased patients had comparable initial scores. Constipation remained unchanged [6] or became worse after mesh [1, 8, 11] or suture [4] rectopexy without sigmoid resection for IRI. We think that the improvement in constipation in the present series may, at least partly, be because of sigmoid resection [12, 13], preservation of the lateral ligaments [26], and rectopexy with suture instead of mesh [30]. For nine out of 12 patients examined in this study, there was a parallel trend towards decrease of colonic transit time and constipation score. In a similar study, using resection rectopexy a significant reduction of colonic transit time was reported [17] in 23 patients. All together, these findings supported that constipation and transit time were related [8, 30, 31]. 

Despite similar anal incontinence scores pre- and postoperatively, the frequency of leakage became reduced from daily to more seldom (Table 4). However, the overall number of patients with incontinence increased, mainly because of rare episodes. Reduction of the rectal intussusception probably improved major continence by reduced dilatation and interference of anal sphincters. On the other hand, both the reduced fecal reservoir following sigmoidal resection and increased age may have slightly deteriorated continence. The latter explanation is supported by demonstration of minor incontinence (gas and solid stool) after mean of 5 years in an age- and parity-matched control group of females without sphincter injury [32]. 

Defecography preoperatively and at short-term postoperatively (Table 2) demonstrated a successful removal and reduction of intussusception and rectocele. The latter has previously been reported for a subgroup of this patient material [16]. In the cohort of 18 patients who had a third defecography at long-term followup the recurrence rate of IRI was 11.1%. Presumably, this figure may be similar to the overall recurrence rate for intussusception in this patient material. Reduced rectal motility seemed to be a transient phenomenon since prevalence from short- to long-term followup was reduced from 16.7% to 5.6%. An alternative method used for obstructed defecation secondary to symptomatic IRI and rectocele is stapled transanal rectal resection (STARR) [33]. The short-term outcome after median 6–20 months in these patients was comparable to our results. However, in one study of 45 patients outcome deteriorated from medium- (18 months) to long-term (42 months) followup, based on symptomatic scores and constipation quality of life [34]. 

The complication rate was comparable to other studies using resection rectopexy [15, 17, 18] or the STARR method [33, 35] for IRI. Rectal perforation was caused by a heat-induced injury from use of ultracision necessitating closure and a temporary ostomy. Partial transection of ureter was treated by suture and temporary stent placement. These patients reported an excellent and improved treatment result, respectively. 

Foremost patients receiving treatment for delayed morbidity or in order to improve outcome reported a decreased satisfaction from short- to long-term followup (Table 5). 

Although, a deep IRI is claimed to cause more severe incomplete evacuation of stool than a moderate intussusception [17], we were in this study unable to demonstrate this relationship. 

Four patients with either recurrent intussusception (*n* = 2) at long term or reduced size of the intussusception (*n* = 2) at short term, but devoid of prolapse at long term, reported improved result for three and much worse result for one of the two latter patients. Accordingly, sigmoid resection per se also must have an impact on the treatment result. Another learning was that detection of small intussusception may be overlooked in one of two consecutive defecographies.

Based on our experience, caution should be made towards operating patients with IRI and concomitant spastic anus or spastic pelvic floor syndrome. These patients have a chronic problem of obstructed defecation due to lack of relaxation of the puborectal sling and should receive biofeedback therapy for training in normal defecation [36]. In this situation, the intussusception may then be of minor significance for rectal emptying.

We used the short-form 36 questionnaire to study HRQoL after resection rectopexy, because normative scores from the background population of Norway were available [29]. This was mandatory since lack of preoperative data on HRQoL in these patients excluded a more optimal comparison of scores before and after surgery. Conclusive age and gender-adjusted data were precluded owing to the limited number of patients. HRQoL was reduced compared with the normal population (Table 6) concerning physical functioning, role limitations due to physical problems, bodily pain, and general health perception. Presumably, factors that reduce HRQoL in these four dimensions are obstructed defecation, anal incontinence, and pain. Despite that two patients had Help syndrome or anorexia nervosa, the general impression was that HRQoL was not deteriorated by alterations in mental health, role limitations due to emotional problems, social functioning, and vitality.

## 5. Conclusion

Ligament preserving resection rectopexy with suture resulted in persistent improvement in constipation and outcome in patients with IRI, both at short-term and long-term followup. 

## Figures and Tables

**Table 1:** Forty-eight patients with internal rectal intussusception. Data are median (range) except where otherwise stated.

Variable	
Duration of symptoms (years)	7 (1–30)
Number of women/men	44/4
Age (years)	53 (29–77)
Followup (months)	
Short term	6 (2–44)
Long term	76 (57–129)

**Table 2:** Defecography preoperatively and short-term postoperatively in 46 of the 48 patients included in this study. Figures are number of patients.

Variable	Preoperatively	Postoperatively
Internal rectal intussusception	46	4
Mucosal prolapse	0	2
Rectocele	22	12
Enterocele	7	4
Reduced rectal motility	0	5
Anismus	1	1
Slight incontinence	3	4

**Table 3:** Reduction in the number of afflicted patients and symptom severity after resection rectopexy for IRI in 48 patients using the KESS score. The first line specifies the number of patients with each symptom. The score for each symptom is given as mean (95% CI).

	Number of patients score		
Variable	Preoperatively	Postoperatively	
		Short term	Long term
Feeling incomplete evacuation	46 3.16 (2.86 to 3.46)	37 1.68 (1.29 to 2.08)	35 1.72 (1.33 to 2.12)
Bloating	39 1.56 (1.21 to 1.90)	29 0.87 (0.58 to 1.16)	35 0.89 (0.68 to 1.10)
Minutes in lavatory/attempt	36 1.27 (0.99 to 1.54)	28 0.81 (0.56 to 1.06)	19 0.47 (0.26 to 0.69)
Abdominal pain	34 1.27 (0.92 to 1.61)	22 0.70 (0.42 to 0.99)	22 0.75 (0.45 to 1.04)
Unsuccessful evacuatory attempts	26 1.22 (0.87 to 1.58)	16 0.52 (0.26 to 0.77)	10 0.31 (0.13 to 0.49)
Enemas/digitation	29 1.50 (1.04 to 1.95)	14 0.70 (0.32 to 1.09)	17 0.79 (0.39 to 1.18)
Laxative use	25 0.97 (0.67 to 1.28)	15 0.43 (0.23 to 0.64)	17 0.60 (0.35 to 0.85)
Painful evacuation effort	22 0.88 (0.52 to 1.18)	12 0.31 (0.13 to 0.49)	5 0.16 (0.02 to 0.30)
Stool consistency	22 1.10 (0.72 to 1.48)	22 0.70 (0.43 to 0.98)	18 0.68 (0.38 to 0.98)
Frequency of bowel movement	10 0.47 (0.18 to 0.77)	6 0.16 (0.02 to 0.30)	3 0.10 (−0.01 to 0.22)

Total score	13.20 (11.41 to 15.00)	6.91 (5.29 to 8.54)	6.35 (4.94 to 7.76) (*P* < 0.0001)

All symptoms were significantly reduced except for stool consistency.

**Table 4:** Symptoms of anal incontinence in 48 patients preoperatively (left), at short-term (middle), and long-term followups (right). The figures are the number of patients.

	Never	Rarely	Sometimes	Weekly	Daily
Incontinence for solid stool	38/40/40	0/1/3	1/3/2	4/3/2	5/1/1
Incontinence for liquid stool	37/34/29	2/5/8	0/3/3	2/3/7	7/3/1
Incontinence for gas	37/33/35	0/2/5	3/1/3	2/8/1	6/4/4
Alteration in lifestyle	35/36/33	0/1/3	2/1/4	1/2/3	10/8/5
				Yes	
Need to wear a pad or plug				10/10/12	
Taking constipating medicines				0/1/3	
Able to delay defecation for 15 min (*n* = 20)				39/42/39	

Rarely and sometimes mean one and two to three episode(s) of anal incontinence during 4 weeks, respectively.

**Table 5:** Report of 48 patients concerning the treatment result at followup. Figures are number of patients, percentages in parenthesis.

	Short term	Long term
Excellent	4 (8.3)	5 (10.4)
Improved	37 (77.1)	31 (64.6)
Unaltered	4 (8.3)	6 (12.5)
Worse	2 (4.2)	5 (10.4)
Much worse	1 (2.1)	1 (2.1)

**Table 6:** HRQoL (SF-36) results for 48 patients operated for IRI at long-term followup versus the general Norwegian population. Scores are given as mean (SD).

Health dimension	Patients	General population	*P* value
	(*n* = 48)	(*n* = 2323)	
Physical functioning	82.5 (17.8)	88.4 (17.4)	0.03
Role limitation due to physical problems*	69.7 (33.0)	79.7 (34.4)	0.04
Role limitation due to emotional problems*	86.3 (24.5)	82.5 (31.8)	0.29
Bodily pain	62.6 (28.1)	75.7 (25.6)	0.002
Social functioning	79.4 (28.1)	86.1 (21.8)	0.18
Mental health*	78.9 (16.8)	78.8 (16.4)	0.97
Vitality*	55.7 (24.8)	60.3 (20.7)	0.21
General health perception	64.8 (24.3)	77.4 (21.8)	0.008

*Although there are subtle differences in the different versions of the SF-36 questionnaire concerning these four health dimensions, the data can be compared.
